# Analysis of Pru av 1.0101 protein expression in *Prunus avium* cultivars with ELISA and liquid chromatography/mass spectrometry

**DOI:** 10.1186/2045-7022-4-S2-P5

**Published:** 2014-03-17

**Authors:** Martie Verschuren, Edward Knaven, Shanna Bastiaan-Net, Kay Foetisch, Stephan Scheurer, Theo Noij, Harry Wichers

**Affiliations:** 1 Avans University of Applied Science, Research group Analysis Techniques in Life Science, Breda, Netherlands; 2 Wageningen UR, Food & Biobased Research, Wageningen, Netherlands; 3 Paul-Ehrlich Institut, Division of Allergology, Langen, Germany

## Background

Food allergy to sweet cherry in Europe is frequently associated with birch pollinosis. After ingestion of fresh cherries, oral allergy symptoms occur due to the cross reactivity of IgE to the birch pollen Bet v 1 homologous cherry protein Pru av 1. In order to detect natural occurring cherry cultivar variations, Pru av 1 protein expression needs to be analysed. However no commercial assays are available so far. Therefore, we determined the protein expression of isoallergen Pru av 1.0101 in five cherry cultivars using ELISA and liquid chromatography-tandem mass spectrometry (LC-MS/MS).

## Method

Total protein was extracted in triplicate from five sweet cherry cultivars (peel and flesh), and the total protein concentration was determined. Pru av 1.0101 protein expression was measured in total cherry protein extracts using a Pru av 1.0101 specific sandwich ELISA. Furthermore, after trypsin digestion of the total protein extract, LC-MS/MS was used to detect specific peptides selective for the Pru av 1.0101 protein. Finally, ELISA and LC-MS/MS results for Pru av 1.0101 were compared.

## Results

Pru av 1.0101 protein could easily be detected in all five cherry cultivars with both ELISA and LC-MS/MS. The sandwich ELISA showed a fivefold difference in Pru av 1.0101 protein expression amongst the five tested cultivars (tested in triplicate), reaching from 22 to 111 µg Pru av 1.0101/g cherry cultivar. In the LC-MS/MS, the most prominent Pru av 1.0101 peptide had a m/z value of 564.8 Da ([M+2H]^2+^). The most abundant fragment ion of this peptide had a m/z value of 758.4 Da ([M+H]^+^). The peak area of this fragment ion was used to compare the differences between Pru av 1.0101 expression in all five cherry cultivars. This LC-MS/MS analysis also showed a five fold difference in Pru av 1.0101 protein expression amongst the tested cultivars. Normalised ELISA and LC-MS/MS data were comparable in all tested cherry cultivars.

## Conclusion

A fivefold difference in Pru av 1.0101 allergen expression can be detected in different cherry cultivars with both ELISA an LC-MS/MS. In the future, this could lead to the identification of natural hypoallergenic cherry cultivars.

